# Primary sclerosing cholangitis complicated with ulcerative colitis and double gene mutations of UGT1A1 and SLC25A13: a case report

**DOI:** 10.3389/fmed.2026.1844455

**Published:** 2026-05-18

**Authors:** Yamei Shi, Kun Chen, Jun Ye, Mei Yang

**Affiliations:** Institute of Gastroenterology of PLA, Southwest Hospital, Army Medical University (Third Military Medical University), Chongqing, China

**Keywords:** genetic testing, primary sclerosing cholangitis, SLC25A13 gene, UGT1A1 gene, ulcerative colitis

## Abstract

**Background:**

Primary sclerosing cholangitis (PSC) is the most common and clinically significant hepatobiliary extraintestinal manifestation of ulcerative colitis (UC), bringing considerable difficulties to clinical diagnosis and treatment and carrying a poor prognosis. Up to now, in-depth research is still required for the pathogenesis, early identification and standardized diagnosis and treatment of PSC-UC overlap syndrome, which holds prominent clinical value.

**Case presentation:**

This report details a case of primary sclerosing cholangitis (PSC) with concurrent ulcerative colitis (UC) in a young female who presented with persistent liver function abnormalities, abdominal pain, and diarrhea. The diagnosis was considered on the basis of histopathology, endoscopy, and imaging findings. Notably, genetic analysis identified heterozygous mutations in UGT1A1 (c.-3275 T > G, c.-1352A > C, c.1352C > T) and SLC25A13 (c.852_855del)—a combination not previously reported in PSC-UC. Treatment with ursodeoxycholic acid, glucocorticoids, and mesalazine led to adequate control of intestinal symptoms but only partial improvement in liver biochemistry.

**Conclusion:**

This case reports a rare instance of primary sclerosing cholangitis complicated with ulcerative colitis in a young female, who was detected to have heterozygous mutations in the UGT1A1 and SLC25A13 genes. These findings generate the hypothesis that genetic susceptibility in the pathogenesis of PSC-UC and may guide future clinical approaches.

## Introduction

Primary Sclerosing Cholangitis (PSC) is a rare progressive liver disease characterized by chronic cholestasis. Currently, there is a lack of effective drug treatment regimens in clinical practice, and liver transplantation remains the only curative method to improve the prognosis (1). Epidemiological studies have shown that approximately 70% of PSC patients are complicated with inflammatory bowel disease (IBD), among which the prevalence of ulcerative colitis (UC) is as high as 55.9%, and the prevalence of Crohn’s disease (CD) is 11.7%. The coexistence of the two diseases tends to increase the complexity of the condition (2). In addition, there is a significant gender distribution difference in PSC patients, with males being the majority (3). However, young female patients are relatively rare in clinical practice, and studies on their disease phenotypes and diagnosis and treatment characteristics still need to be supplemented, which provides a unique perspective for this case report.

The clinical manifestations of PSC are insidious, often presenting with abnormal liver function and cholestasis-related symptoms (such as pruritus and fatigue) (4), while the complicated UC mostly presents with intestinal symptoms such as abdominal pain, diarrhea, and hematochezia (5). The overlapping characteristic of such “hepato-biliary-intestinal” dual symptoms easily leads to the masking of PSC symptoms by UC. In addition, the imaging and biochemical manifestations of PSC overlap with those of other chronic liver diseases. Clinical diagnosis relies on cholestatic biochemical indicators (such as elevated alkaline phosphatase and *γ*-glutamyl transpeptidase) and the characteristic of multifocal bile duct stenosis shown by magnetic resonance cholangiopancreatography (MRCP), which further increases the difficulty of dual diagnosis (6–8). Especially in the rare patient group of young women, the diagnosis and treatment are easily delayed due to insufficient clinical vigilance.

It is known that the UGT1A1 gene regulates bilirubin metabolism, and the SLC25A13 gene is involved in the maintenance of hepatocyte mitochondrial function. Mutations in both genes are closely related to the genetic susceptibility to hepatobiliary diseases (9, 10). However, this combination of double gene mutations has not been found in PSC patients complicated with UC before. This case is a 26-year-old female, which not only conforms to the rare gender characteristic of PSC complicated with UC, but also its genetic testing results and treatment response (intestinal symptoms improved after standardized treatment, but liver function indicators were poorly controlled). We hypothesize that this combination of double gene mutations may affect the response of liver lesions to conventional treatment by influencing hepatocyte metabolism and bile duct injury repair process. This case generates a testable hypothesis regarding the genetic pathogenesis of PSC-UC and a potential molecular marker for clinical judgment of the prognosis of such patients.

In view of the limited current treatment methods for PSC (mainly symptomatic support), the report of this case can not only enrich the genetic variation spectrum of PSC-UC patients, but also promote the academic attention to the correlation between “genetic factors—disease phenotype -treatment response.” It provides a new screening idea of “alerting to rare gene mutations” for the diagnosis of young female PSC-UC patients, and at the same time provides a reference for the formulation of subsequent individualized treatment plans and mechanism research.

## Case report

A 26-year-old female was admitted for abnormal liver function (>1 year) and diarrhea/abdominal pain (1 month). She denied alcohol use, fatty liver, parasitic infection, chronic diseases, or surgery, but had a history of lumbosacral pain. In 2023, she took Guipi Mixture, Xuefu Zhuyu Oral Liquid, and progesterone capsules for COVID-19, influenza A, and irregular menstruation (dosage/course unknown). In October 2023, elevated transaminase was found; abdominal MRI/CT suggested cholestasis, so she received ursodeoxycholic acid + glutathione. December 2023 autoantibody review showed ANA 1:100 and anti-plasmacyte liver antigen 1 antibody (+), with no treatment adjustment.

**Figure 1:**
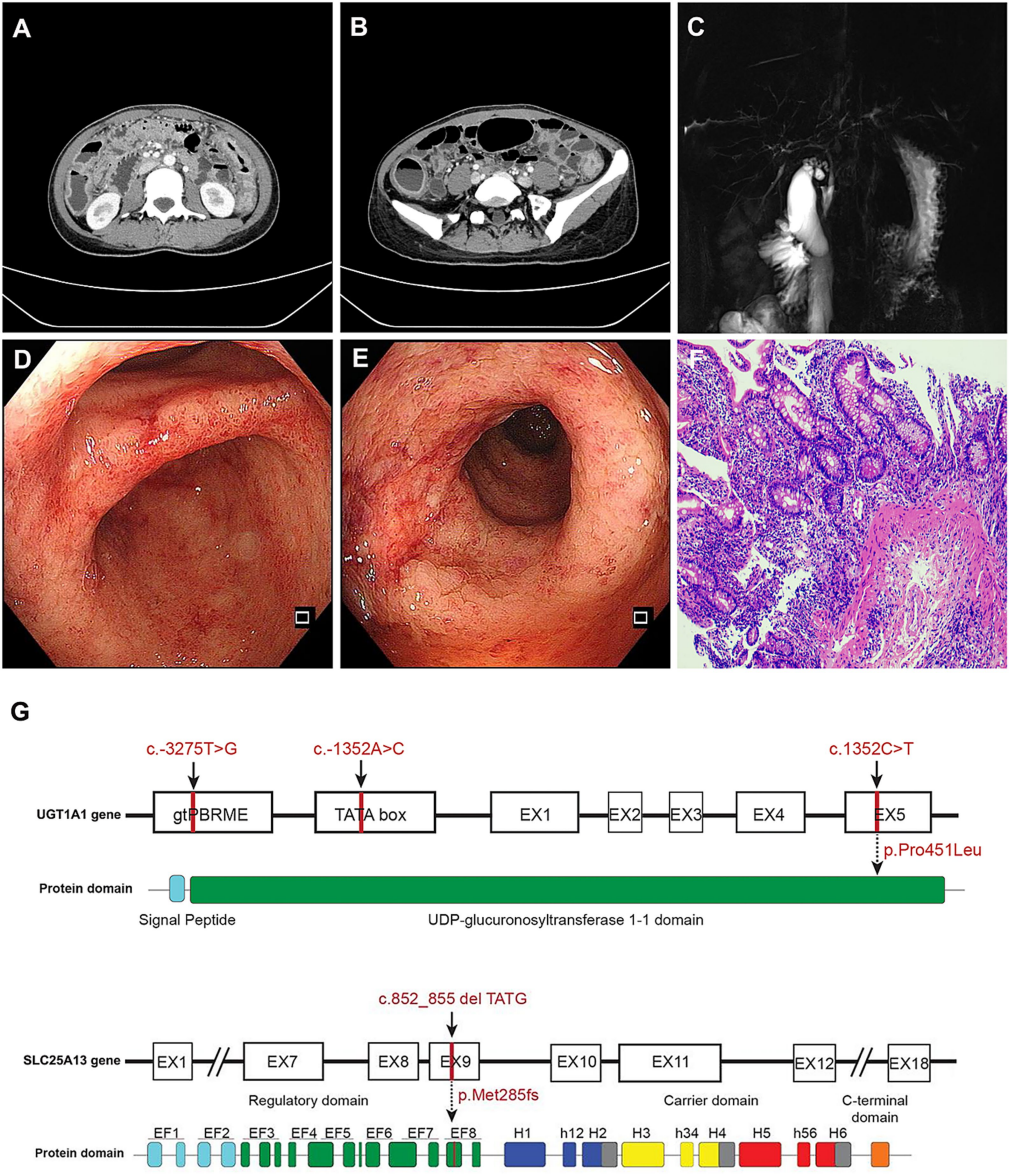
Abdominal CT, MRCP, colonoscopy findings, intestinal histopathological features, and high-throughput sequencing for genetic testing of the patient. **(A,B)** Show abdominal CT images. **(C)** Displays biliary duct changes on MRCP. **(D,E)** Present intestinal ulcerative colitis, with **(D)** depicting the ileocecal region and **(E)** the descending colon. **(F)** Refers to the pathological biopsy result. **(G)** Schematic diagram of *UGT1A1* and *SLC25A132* gene mutation sites.

In July 2024, she had abdominal distension and unformed stools (no treatment). September 2024 liver biopsy showed chronic moderate hepatitis (Supplementary Figure 1). Autoimmune hepatitis-related autoantibodies were all negative, and serum IgG level was normal, which excluded autoimmune hepatitis (Tables 1, 2). By late January 2025, she had diarrhea; early February brought postprandial abdominal pain, night sweats, and fatigue, and she was admitted on March 8 for hematochezia. On admission, BMI was 18.34 kg/m^2^ (underweight); no icterus, edema, or abdominal tenderness; 2 kg weight loss in 1 year. Etiological/immunological tests were normal; stool culture showed no pathogens. Abdominal CT suggested UC (Figures 1A,B); MRCP showed bile duct rigidity, dilatation, and thickening (Figure 1C); liver elastography indicated obvious fibrosis. Colonoscopy (Mayo score 3) and pathology supported UC (Figures 1D–F). Genetic testing revealed UGT1A1 (c.-3275 T > G, c.-1352A > C, c.1352C > T) and SLC25A13 (c.852_855del) heterozygous mutations (Figure 1G).

**Table 1:** Summary of autoimmune antibody test results.

Test panel	Test parameter	Result	Reference range
Autoantibody spectrum (IgG)	Anti-smooth muscle antibody (ASMA)	Negative	Negative
	Anti-nuclear antibody (ANA)	Negative	Negative
	Anti-mitochondrial antibody (AMA)	Negative	Negative
	Anti-liver-kidney microsomal antibody (Anti-LKM)	Negative	Negative
	Anti-nuclear ribonucleoprotein/Smith antibody (nRNP/Sm)	Negative	Negative
	Anti-Smith antibody (Sm)	Negative	Negative
	Anti-Sjögren’s syndrome A antibody (SS-A)	Negative	Negative
	Anti-Sjögren’s syndrome B antibody (SS-B)	Negative	Negative
	Anti-topoisomerase I (Scl-70) antibody (Scl-70)	Negative	Negative
	Anti-polymyositis/scleroderma antibody (PM-Scl)	Negative	Negative
	Anti-histidyl-tRNA synthetase antibody (Jo-1)	Negative	Negative
	Anti-centromere protein B antibody (CENP-B)	Negative	Negative
	Anti-proliferating cell nuclear antigen antibody (PCNA)	Negative	Negative
	Anti-double-stranded DNA antibody (ds-DNA)	Negative	Negative
	Anti-nucleosome antibody	Negative	Negative
	Anti-histone antibody	Negative	Negative
	Anti-ribosomal P protein antibody	Negative	Negative
Autoimmune liver disease panel	Anti-mitochondrial M2-3E antibody (M2-3E)	Negative	Negative
	Anti-promyelocytic leukemia antibody (PML)	Negative	Negative
	Anti-liver-kidney microsomal type 1 antibody (LKM-1)	Negative	Negative
	Anti-soluble liver antigen/liver-pancreas antibody (SLA/LP)	Negative	Negative
	Antimitochondrial antibody M2 subtype(AMA-M2)	Negative	Negative
	Anti‑speckled 100 kDa antibody (SP100)	Negative	Negative
	Anti-nuclear envelope glycoprotein 210 antibody (GP210)	Negative	Negative
	Anti-liver cytosol type 1 antibody (LC-1)	Negative	Negative
	Anti-SS-A/Ro 52 kDa antibody (RO-52)	Negative	Negative

**Table 2:** Differential diagnosis of primary sclerosing cholangitis (PSC), autoimmune hepatitis (AIH), and IBD-associated hepatopathy.

Feature	PSC	AIH	IBD-associated hepatopathy
Main injury pattern	Cholestatic	Hepatocellular	Mild or mixed
Typical biochemical changes	Markedly elevated ALP/GGT	Markedly elevated ALT/AST	Mildly abnormal
Autoantibodies	Usually negative or low titer	ANA, SMA, anti-LKM1 often positive	Usually negative
Serum IgG	Normal or mildly elevated	Significantly elevated	Normal
Typical imaging findings	Bile duct strictures and dilatation (MRCP)	No bile duct changes	No bile duct changes
Liver histology	Periductal fibrosis, onion‑skin lesions	Interface hepatitis, plasma cell infiltration	Nonspecific reactive changes
Association with IBD	Closely associated (≈70%)	Less common	Directly related

Based on the biliary imaging features consistent with PSC, clinical evidence of cholestasis, IBD-related intestinal manifestations and histopathological findings, PSC-UC with heterozygous UGT1A1 and SLC25A13 mutations was considered as the clinical diagnosis. The treatment regimen included oral ursodeoxycholic acid capsules (250 mg tid), prednisone acetate tablets (40 mg qd), mesalazine sustained-release granules (1,000 mg tid), and ferrous lactate tablets (0.2 g tid). Adjunctive therapy consisted of calcium carbonate D3 tablets, vitamin D drops, and whole-protein enteral nutritional powder to address nutritional deficiencies and support overall health.

Follow-up: April 2025—intestinal symptoms relieved, anemia uncorrected, cholestasis indices abnormal; May 2025—anemia corrected, cholestasis worsened (azathioprine added); September 2025—cholestasis slightly improved (fenofibrate/bezafibrate/obeticholic acid added), fibrosis alleviated; October 2025—intestinal inflammation remission (Supplementary Figure 2), liver function still elevated (Table 3). Long-term follow-up and treatment optimization are needed.

**Table 3:** Longitudinal liver function tests and immunological markers of the patient.

Parameter	Unit	Reference range	Sep-2024	Mar-25	Apr-25	May-25	Sep-25	Oct-25
Alanine aminotransferase (ALT)	U/L	0–42	114	11.5	13.5	43.1	73.1	47.7
Aspartate aminotransferase (AST)	U/L	0–42	118.4	31.2	21.6	29.9	64.2	44.4
Alkaline phosphatase (ALP)	U/L	34–114	376.4	383.3	121.7	231.9	140.8	124.2
Gamma-glutamyl transferase (GGT)	U/L	4–50	181.6	190	84.1	422.9	193.7	56.8
Total bilirubin	μmol/L	6–21	23.23	20	8.1	11.4	12.7	13.1
Direct bilirubin	μmol/L	0–6	16.44	10.3	1.9	3.3	4.2	3.6
Albumin	g/L	38–51	33	29.6	39.5	41.3	43.3	40.4
Antimitochondrial antibody (AMA)		Negative	Negative	Negative	Negative	Negative	Negative	Negative
Perinuclear anti-neutrophil cytoplasmic antibody (p-ANCA)		Negative	Negative	Negative	Negative	Negative	Negative	Negative

## Discussion

The comorbidity of primary sclerosing cholangitis (PSC) and ulcerative colitis (UC) has attracted increasing clinical attention. This case report describes a 26-year-old female patient, and for the first time reveals the clinical significance of the UGT1A1 and SLC25A13 gene mutation combination in PSC-UC considered on the basis of clinical and imaging findings, which is in sharp contrast to the phenomenon mainly focused on male patients in the literature (3, 11, 12). However, relevant data in China are relatively limited and have not been fully explored in young female patients, so the report of this case has important clinical value and academic significance.

UGT1A1 is the only enzyme capable of metabolizing bilirubin. Mutations in its encoding gene lead to decreased activity of uridine diphosphate-glucuronosyltransferase, resulting in abnormal bilirubin metabolism and increased hepatocyte damage and liver burden (13). SLC25A13 is an autosomal recessive genetic disease. Mi-Ae Jang et al. conducted an analysis of data from 17,501 East Asians, showing a carrier rate of 1/62, and identified 23 pathogenic SLC25A13 variants, among which c.852_855del is the most common. Pathogenic variants of SLC25A13 can cause neonatal intrahepatic cholestasis caused by citrin deficiency (NICCD), and citrin deficiency leads to growth retardation, dyslipidemia, and adult-onset type II citrullinemia (CTLN2) (14). Therefore, we hypothesize that the combined mutation of UGT1A1 and SLC25A13 may lead to bilirubin metabolism disorders and aggravate liver damage, providing a new perspective for the pathological mechanism of PSC, and suggesting that clinicians should pay attention to the relationship between genetic factors and pathological mechanisms (12, 15).

Currently, there is no specific drug for the treatment of PSC. Ursodeoxycholic acid can improve the clinical and biochemical indicators of PSC patients, but cannot improve the outcomes of liver transplantation or death (1). A randomized, double-blind, placebo-controlled phase III trial included 30 PSC patients who received UDCA treatment before the study. They were given 200 mg fenofibrate, and the control group was given placebo, once a day for 6 months. The results showed that ALP was significantly reduced, and the prognosis of PSC patients was better (16). PPAR agonists have the effects of regulating fatty acid metabolism and anti-inflammation. The activation of PPAR can be achieved by using lipid-lowering drugs (such as bezafibrate). Therefore, the combined use of UDCA and fibrates can also improve the patient’s biochemical indicators and pruritus (17). In this case, after treatment with ursodeoxycholic acid, bezafibrate, glucocorticoids, mesalazine combined with symptomatic supportive therapy, the patient’s intestinal symptoms improved significantly, but the liver function indicators remained poorly controlled, which suggests that clinicians should focus on the impact of genetic factors when facing similar cases.

Young female patients with PSC-UC represent a rare and understudied subgroup with atypical clinical manifestations, in whom intestinal symptoms frequently mask biliary tract involvement and lead to delayed diagnosis (18). Moreover, when PSC patients are complicated with IBD, their clinical manifestations and prognosis are significantly different, and they may face a higher risk of complications, including increased incidence of liver function impairment and colorectal cancer (19, 20). Genetic testing for UGT1A1 and SLC25A13 variants helps identify potential susceptibility factors and clarify disease mechanisms, while individualized treatment regimens and long-term monitoring are essential to optimize liver biochemistry and clinical outcomes given the poor response of liver lesions to conventional therapy. Future research should investigate gene–environment interactions and validate these genetic markers in larger cohorts to improve early recognition, risk stratification, and targeted management of this rare overlap syndrome (21).

This study is limited by its single-case design without mechanistic experiments or control groups; thus, the causal link between the identified variants and PSC-UC pathogenesis remains to be confirmed. Nevertheless, this case expands the clinical and genetic spectrum of PSC-UC and underscores the importance of comprehensive evaluation and personalized care in young female patients.

### Limitations

This study is a single case report without in-depth mechanistic experiments or matched control groups, thus failing to verify the specific molecular mechanisms of action of genetic variants and their causal association with the disease.

## Conclusion

This study reports a rare case of PSC-UC considered on the basis of clinical and imaging findings in a young female with specific genetic variants. Conventional therapy controlled intestinal symptoms but not liver function, supporting the hypothesis that genetic factors may affect PSC-UC pathogenesis and treatment response. These findings highlight the value of genetic testing for this patient group and enrich PSC-UC’s clinical and genetic spectrum.

## Data Availability

The datasets presented in this study can be found in online repositories. The names of the repository/ repositories and accession number(s) can be found below: https://ngdc.cncb.ac.cn/. BioProject [PRJCA052118], OMIX [OMIX013291].

## Supplementary material

SUPPLEMENTARY FIGURE 1 Pathological biopsy features of the patient's liver tissue. **(A–C)** Consistent with the features of chronic moderate hepatitis.

SUPPLEMENTARY FIGURE 2 **(A–H)** Follow-up colonoscopy findings after treatment.
