# Supplementary figures and images for: Primary sclerosing cholangitis complicated with ulcerative colitis and double gene mutations of UGT1A1 and SLC25A13: a case report
Source: Front Med (Lausanne). 2026 May 18;13:1844455. doi: 10.3389/fmed.2026.1844455 (PMC13222816; doi:10.3389/fmed.2026.1844455)

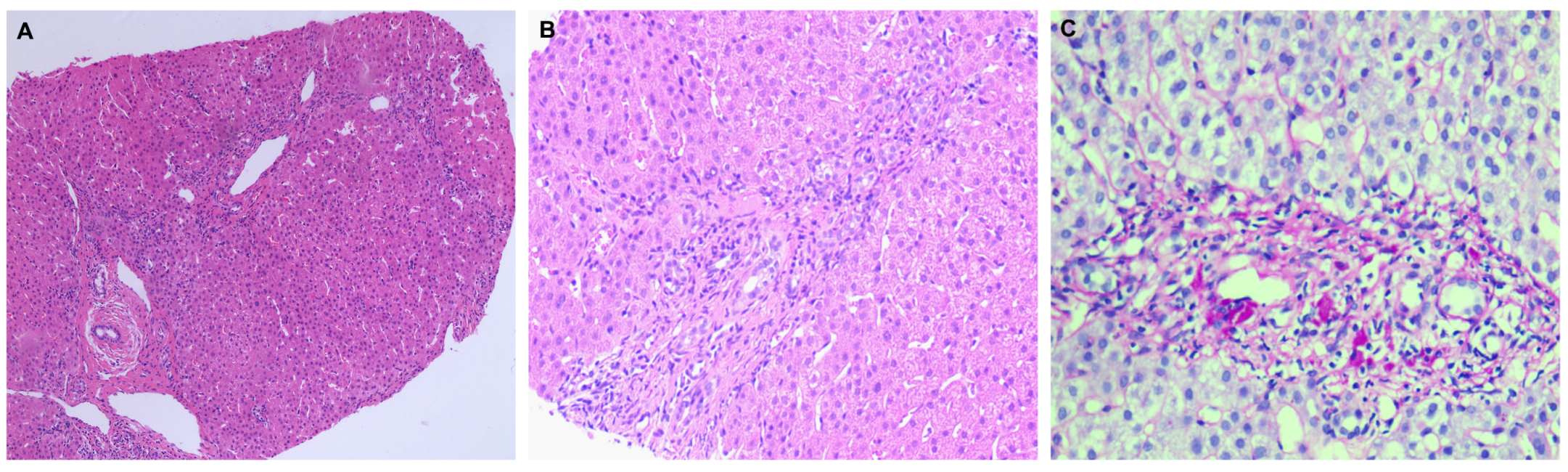

Supplement: SUPPLEMENTARY FIGURE 1 — Pathological biopsy features of the patient's liver tissue. (A–C) Consistent with the features of chronic moderate hepatitis. [file Image_1.tif]

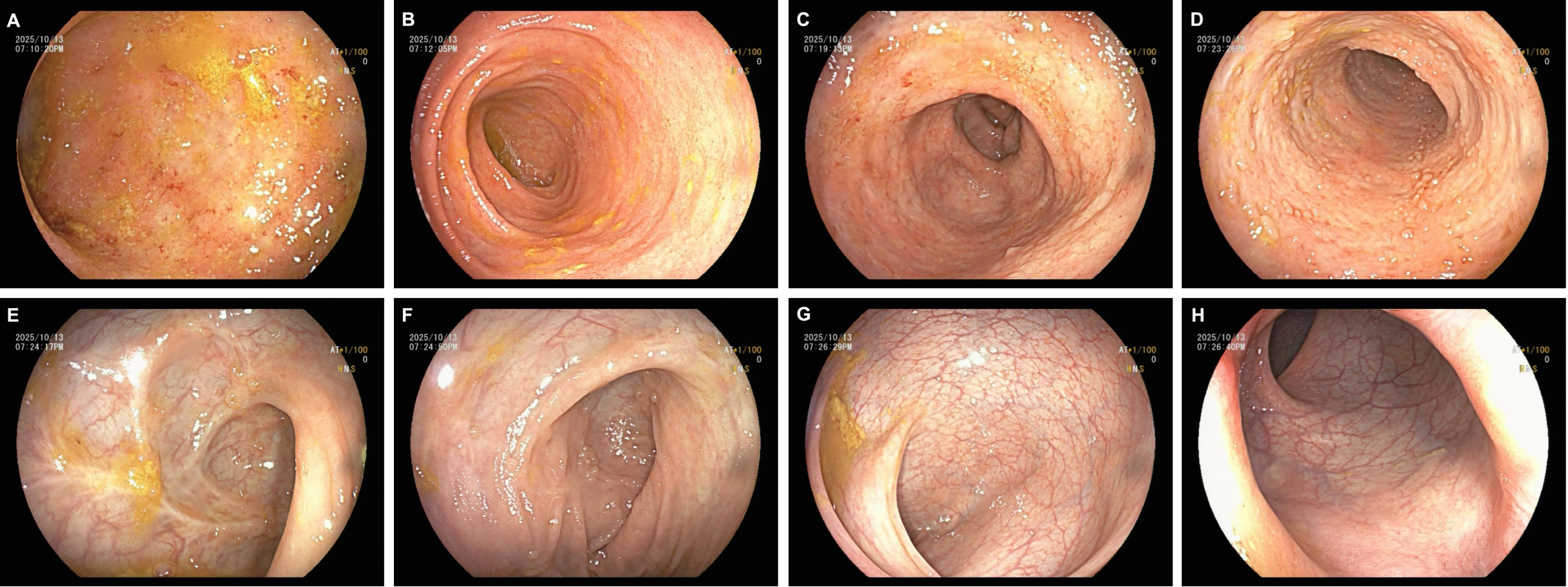

Supplement: SUPPLEMENTARY FIGURE 2 — (A–H) Follow-up colonoscopy findings after treatment. [file Image_2.tif]
